# Supplementary figures and images for: Tissue-specific reprogramming of glutamine metabolism maintains tolerance to sepsis
Source: PLoS One. 2023 Jul 6;18(7):e0286525. doi: 10.1371/journal.pone.0286525 (PMC10325078; doi:10.1371/journal.pone.0286525)

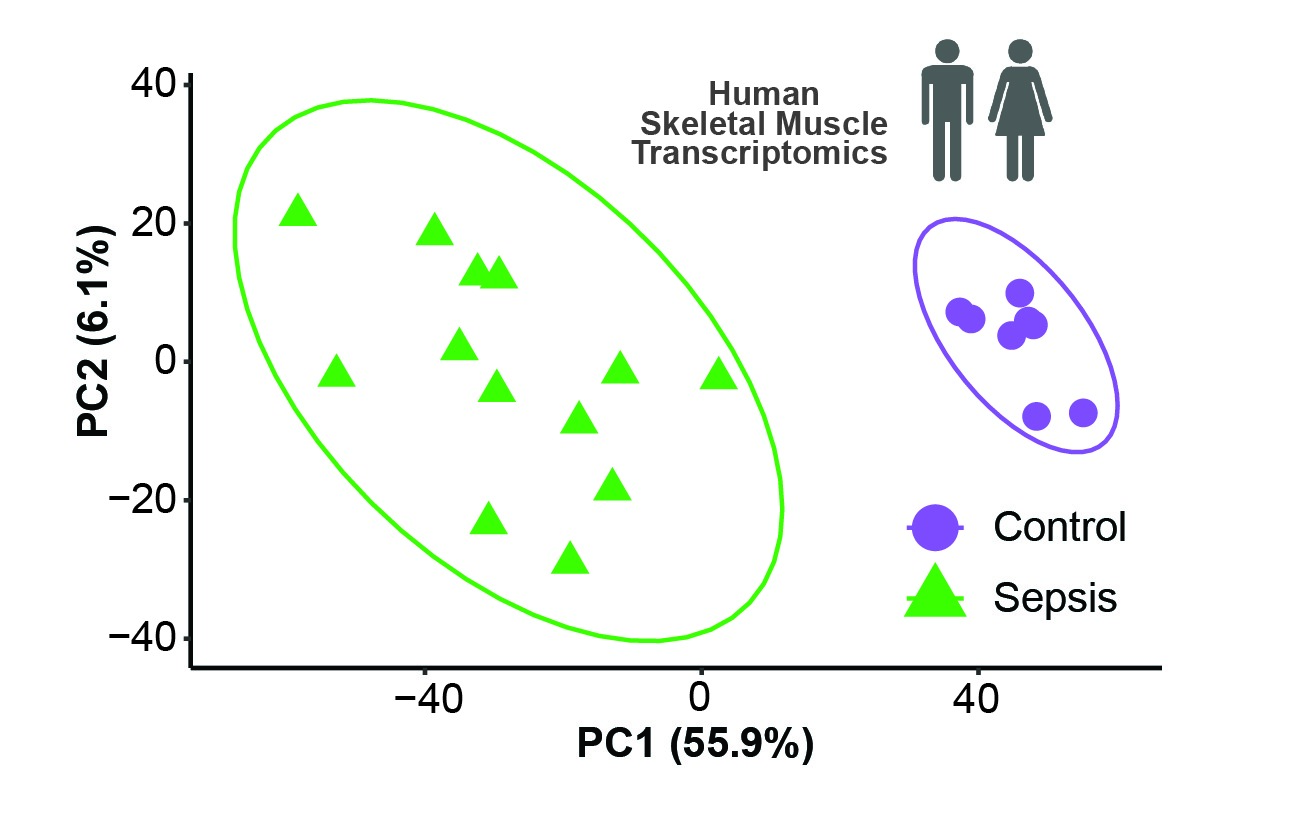

Supplement: S1 Fig — (TIF) [file pone.0286525.s001.tif]

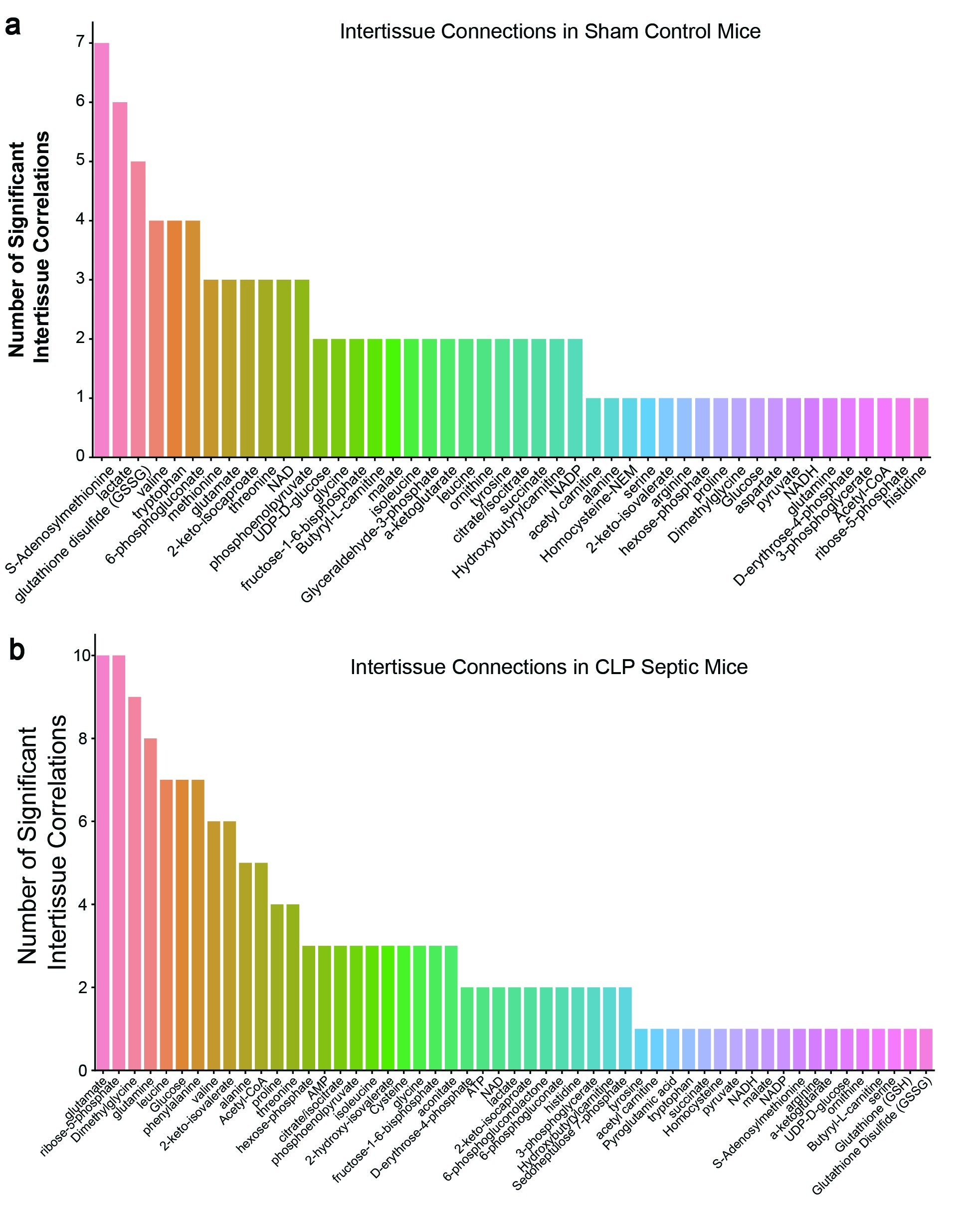

Supplement: S2 Fig — (TIF) [file pone.0286525.s002.tif]

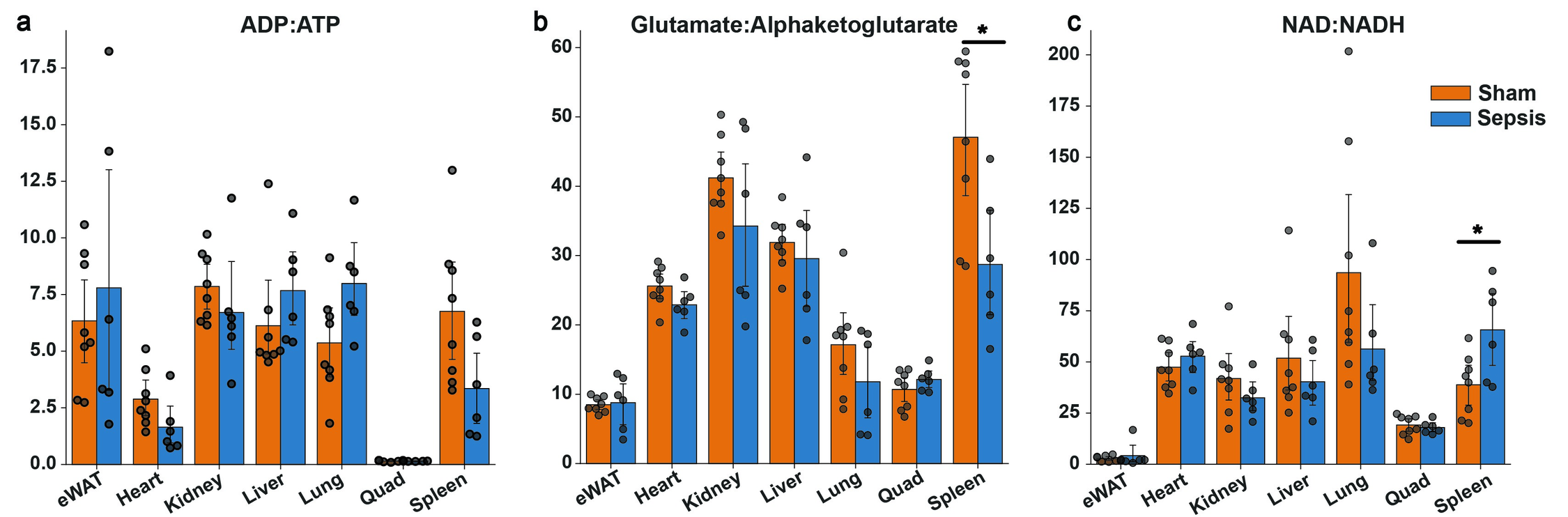

Supplement: S3 Fig — Blue bars are septic mice and orange bars are sham control mice. *p < 0.05 by student’s t-test. (TIF) [file pone.0286525.s003.tif]

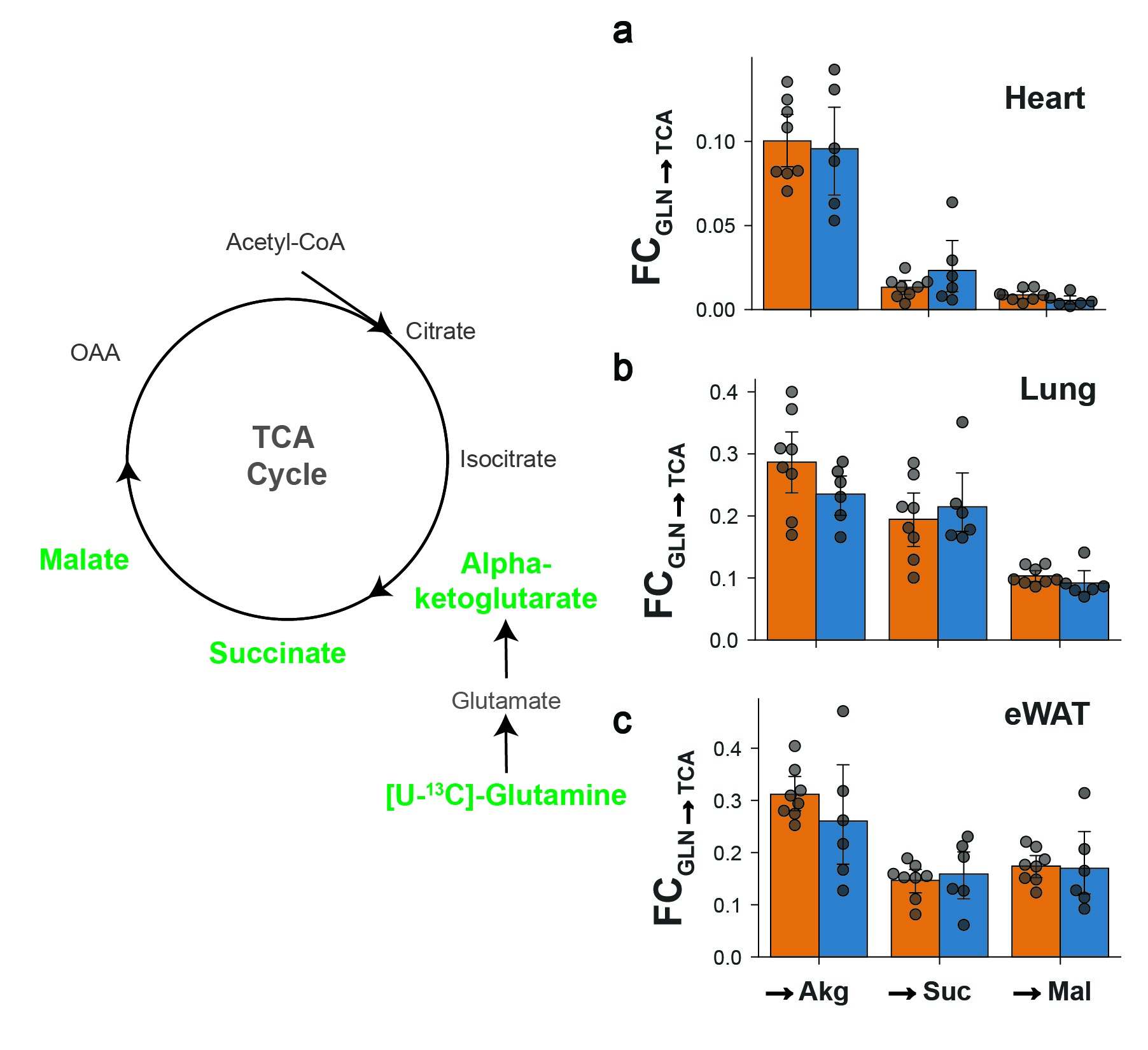

Supplement: S4 Fig — Akg = alphaketoglutarate, Suc = succinate, Mal = malate, GLN = glutamine. Blue bars are septic mice and orange bars are sham control mice. (TIF) [file pone.0286525.s004.tif]

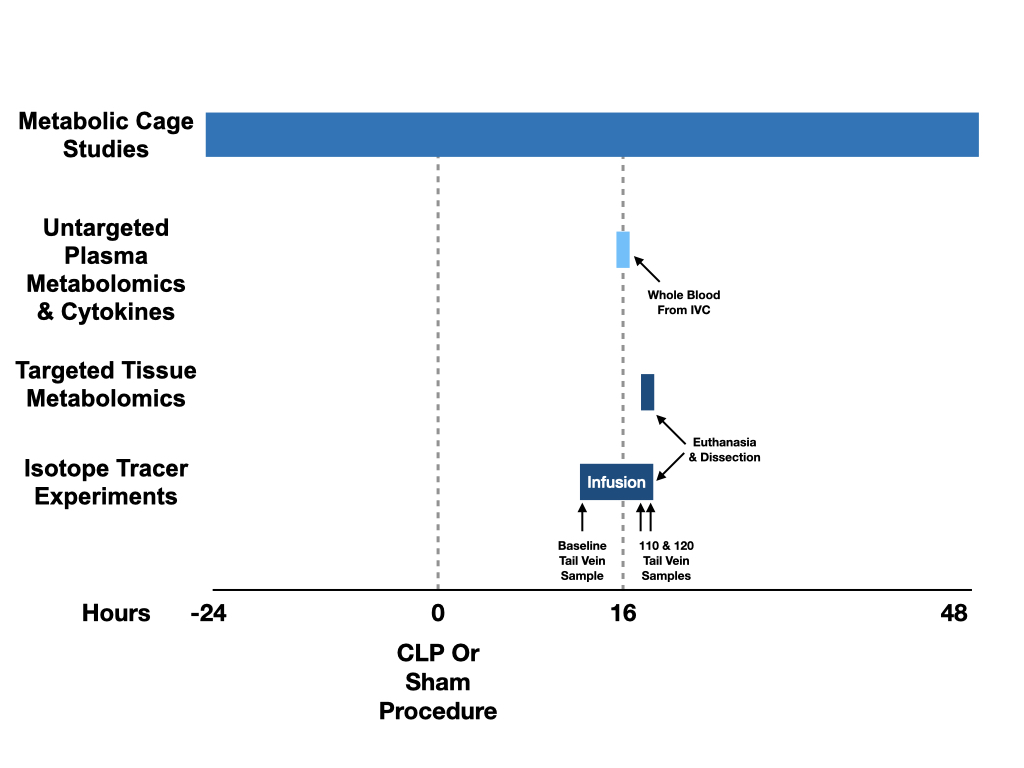

Supplement: S5 Fig — The timeline is given in hours relative to the CLP or Sham procedure (Time 0). The metabolic cage studies were conducted for the 24 hours before, and 48 hours after the procedure. The untargeted metabolomics and cytokine assays were collected 16 hours post-sepsis from the inferior vena cava (IVC). The targeted tissue metabolomics and isotope tracer experiments were performed at the same time, following a 2 hour isotope infusion, ending between 16–17 hours post procedure. Blue bars indicate approximate timing of animal involvement of data collection. (TIF) [file pone.0286525.s005.tif]
